# Prodigiosin in glioblastoma: mechanistic pharmacology and rationale for its development as a radiosensitiser

**DOI:** 10.3389/fphar.2026.1767203

**Published:** 2026-06-30

**Authors:** Hansika Ray

**Affiliations:** School of Biological Sciences, University of Manchester, Manchester, United Kingdom

**Keywords:** autophagy-associated cell death, DNA damage response, drug discovery, glioblastoma, glioma stem cells, marine-derived compounds, prodigiosin, radiosensitiser

## Abstract

**Background:**

Glioblastoma (GBM) exhibits marked resistance to radiotherapy due to hypoxia, metabolic adaptation, enhanced DNA damage response, and the persistence of glioma stem cells (GSCs). Radiosensitisers have therefore become a key therapeutic focus, yet clinically effective agents remain limited.

**Objective:**

This narrative review synthesises current knowledge on GBM radioresistance mechanisms and evaluates prodigiosin (PG)–a marine-derived tripyrrole pigment–as a potential radiosensitiser, based on its diverse antitumour mechanisms.

**Content:**

PG demonstrates multifaceted cytotoxic activity in GBM through cytosolic acidification, mitochondrial destabilisation, ER stress and autophagy-associated cell death, DNA intercalation and copper-dependent oxidative cleavage, modulation of MAPK and PI3K–Akt signalling, and inhibition of proliferative and survival pathways. These actions intersect with major determinants of radioresistance, including DNA repair efficiency, ROS adaptation, GSC maintenance and checkpoint recovery. We outline mechanistic hypotheses for PG–radiation synergy, discuss delivery challenges such as BBB penetration, and propose a structured roadmap for *in vitro*, *in vivo* and translational investigation.

**Conclusion:**

Although no studies have directly evaluated PG in combination with radiation, its biological profile supports strong theoretical potential as a radiosensitiser. This review integrates current evidence into a mechanistic pharmacology framework and outlines a structured experimental roadmap for evaluating prodigiosin as a marine-derived radiosensitiser in preclinical drug discovery.

## 1 Introduction

Glioblastoma multiforme (GBM) is the most aggressive and malignant form of glioma and is categorised as Isocitrate Dehydrogenase-wildtype (IDH-wt) diffuse astrocytic (primary) tumour, the highest grade in the World Health Organisation (WHO) 2021 (Kanderi et al., 2022; Khabibov et al., 2022; Wirsching et al., 2016). In adults, it arises either *ab initio* or from lower-grade astrocytomas, most frequently in the frontal or temporal lobes (Colopi et al., 2023; Thakkar et al., 2014; Grochans et al., 2022). GBM represents the most prevalent primary malignant brain tumour in adults and accounts for 45.2% of cases, although its yearly incidence is 3.1 per 100,000, which is low in comparison to other cancer types (Kanderi et al., 2022; Hanif et al., 2017; Su and Abdullah, 2016). It is a catastrophic brain cancer with 15–18 months of median survival, falling to under 6 months if left untreated, with a 5-year survival rate of just 5.5% post-diagnosis (Kanderi et al., 2022; Hanif et al., 2017; Su and Abdullah, 2016).

IDH-wt GBM develops *de novo*, accounts for ∼90% of cases and often arises in older individuals (Hanif et al., 2017). GBM is notorious for its poor prognosis, primarily driven by both intertumoural and intratumoural heterogeneity (Becker et al., 2021). Due to this highly intricate, multifaceted and heterogeneous nature along with elevated chances of recurrence post-standard multimodal treatment, GBM remains incurable. The current standard of GBM management is surgery, followed by chemotherapy or radiotherapy (Rong et al., 2022). Each treatment causes damage to both the normal and tumour cells, with the normal cells recovering between treatment sessions unlike tumour cells.

Patients undergoing chemotherapy are provided with temozolomide (TMZ) as the first-line agent (Gilard et al., 2021). TMZ is an oral alkylating agent which causes DNA methylation to disrupt cell proliferation and is especially effective against GBM with minimal or zero expression of O (6)-methylguanine-DNA methyltransferase (MGMT) (Chien et al., 2021). These treatments have been demonstrated to increase survival rates and enhance patient outcomes. Nevertheless, radioresistance remains a critical concern in GBM treatment. Radioresistance arises from several factors operating in unison–tumour microenvironment (TME), cellular energetics reprogramming, tumour heterogeneity, cell cycle regulation, DNA damage and repair and microRNAs (miRNAs) (Ali et al., 2020).

Prodigiosin (PG) is a red tripyrrole pigment of the prodiginine family, a secondary metabolite secreted by *Serratia marcescens* and other Gram-negative bacteria. It induces apoptosis across diverse malignancies–and in GBM cell lines reduces cell viability dose-dependently while depleting the self-renewing neurosphere population at doses far below those required of temozolomide. This GBM-active cytotoxicity, combined with a pleiotropic mechanistic profile that intersects the principal determinants of radioresistance, makes PG of particular interest as a candidate radiosensitiser–the rationale developed in this review. Rather than providing experimental validation, this review aims to synthesise existing mechanistic evidence and propose a rational drug discovery framework to guide preclinical evaluation of prodigiosin–radiotherapy combinations in glioblastoma.

## 2 Radioresistance in GBM

### 2.1 TME and hypoxia

Hypoxia is a primary characteristic of solid tumours since the rapid growth of tumours surpasses neovascularisation, resulting in heterogeneous oxygen diffusion to all tumour zones (Ali et al., 2020). Hypoxia-inducible factors (HIFs) regulate the tumourigenic potential of glioma stem cells (GSCs) (Ali et al., 2020; Li et al., 2009). Reactive oxygen species (ROS) such as hydroxyl radical, hydrogen peroxide and superoxide ion, predominantly facilitate DNA damage induced by radiotherapy under normoxia (Ali et al., 2020). However, the capacity of radiotherapy to induce oxidative stress (generated by free radicals), declines under hypoxic conditions, illustrating the mechanism of hypoxia-induced radioresistance (Ali et al., 2020). Moreover, a study demonstrated that hypoxia-mediated modulation of the functional crosstalk between HIF-α, extracellular signal-related kinases (ERKs) and DNA-dependent protein kinase catalytic subunit (DNA-PKcs) also induces GBM radioresistance (Marampon et al., 2014). Hypoxia also promotes radioresistance by enhancing stemness of GSCs–hypoxia activation leads to HIF-2α-mediated Octamer-binding Transcription Factor 4 (OCT4) activation, thereby regulating the self-renewal and multipotency potential of GSCs (Li et al., 2009; Li et al., 2013).

While hypoxia has been established as a crucial factor contributing to radioresistance in GBM, it represents only one aspect of the complex TME which also plays a central role in tumour proliferation and progression (Lorger, 2012). Research has suggested that TME niches provide GSCs various mechanisms to impede radio- and chemotherapies (Fidoamore et al., 2016). For instance, pathways such as Wnt/β catenin, Notch and Sonic Hedgehog (SHH) involved in chemo- and radioresistance. Wnt signalling-associated genes are upregulated in radioresistant GBM cells–overexpression of Wnt/β catenin has been associated with the aggressiveness and poor prognosis of GBM (Ali et al., 2020). Furthermore, Notch is another pathway that is intricately involved in radioresistance via Phosphatidylinositol 3-Kinase–Protein Kinase B (PI3K-Akt) regulation (Ali et al., 2020).

### 2.2 Glioma stem cells and cellular plasticity

Radioresistant Glioma initiating cells (GICs), specifically CD133+ GICs, activate DNA damage checkpoint kinases Chk1 and Chk2 in response to irradiation (Bao et al., 2006). CD133+ GICs repair radiation-induced DNA damage more effectively than the CD133-cells, which underlies their capacity for radioresistance (Bao et al., 2006). It has also been shown that GIC radioresistance also operates via overexpression of Proliferating Cell Nuclear Antigen-associated factor (PAF) which associates with PCNA to release Translesion Synthesis DNA Polymerase Eta (TLS Pol η), following the irradiation of GICs, leading to error-free DNA synthesis restoration and therefore GSC proliferation and radioresistance (Sek et al., 2017). Furthermore, tumour heterogeneity is majorly associated with GSCs in the TME (Yaes, 1989). GSCs and GICs (CD133+) contribute to GBM radioresistance through intrinsic overactivation of PI3K-Akt and PTEN pathways and hyperactivation of DNA damage checkpoint pathways (Bao et al., 2006; Castellino and Durden, 2007). Concurrent STAT3 and ERK1/2 signalling further sustains the radioresistant phenotype, and acquired radioresistance in GBM is mediated in part by SOCS1/3 dysregulation (Xie et al., 2019; Ventero et al., 2019). This intratumoural heterogeneity–distinct molecular and genetic differences of the different cells within a tumour–results in contrasting responses to radiotherapy. It has also been demonstrated that treatment with radiation, made radioresistant populations highly dominant, elevating the overall tumour resistance, contributing to the poor prognosis of GBM (Ali et al., 2020; Bao et al., 2006).

### 2.3 DNA damage response and repair pathways

Double strand break (DSB) repair is induced by HDAC-4 and -6 to promote radioresistance (Marampon et al., 2017; Golding et al., 2009). Overexpression of EGFR variant III (EGFRvIII) also independently generates radioresistance in GBM cells by activating non-homologous end joining (NHEJ) and homologous recombination (HR) (Marampon et al., 2017; Golding et al., 2009). Another crucial signalling pathway, PI3K-Akt, is commonly abnormally activated and has been correlated with GBM poor prognosis and patient outcomes (Ali et al., 2020). In GBM, elevated AKT expression and activity promotes tumour progression, recurrence and radioresistance. AKT activation enhances DNA damage response (DDR) efficiency by accelerating Phosphorylated Histone H2AX (Ser139) (γ-H2AX) foci resolution in irradiated GBM cells and AKT repression mediates unrepairable DSBs in irradiated U251 GBM cells (Golding et al., 2009). Tumour suppressor Leucine-Rich Repeats and Immunoglobulin-Like Domains Protein 1 (LRIG1) has been shown to modify radioresistance by regulating the Akt pathway. On irradiation, it is downregulated contributing to radioresistance since its upregulation markedly decreases EGFR signalling and AKT phosphorylation by heightening DNA damage and radiosensitivity (Yang et al., 2015). Moreover, PTEN loss or mutation is predilect in GBM and leads to Akt activation resulting in radioresistance (McCabe et al., 2015). Beyond radiotherapy, combination strategies targeting both EGFR and DNA repair pathways have also demonstrated efficacy against GSC-driven temozolomide resistance (Sharifi et al., 2019).

Ionising radiation is known to induce autophagy instead of apoptosis in the GBM cells, as a cytoprotective strategy (Aiyappa-Maudsley et al., 2022). This autophagic response allows tumour cells to survive radiation-induced stress, further reinforcing radioresistance.

**FIGURE 1:**
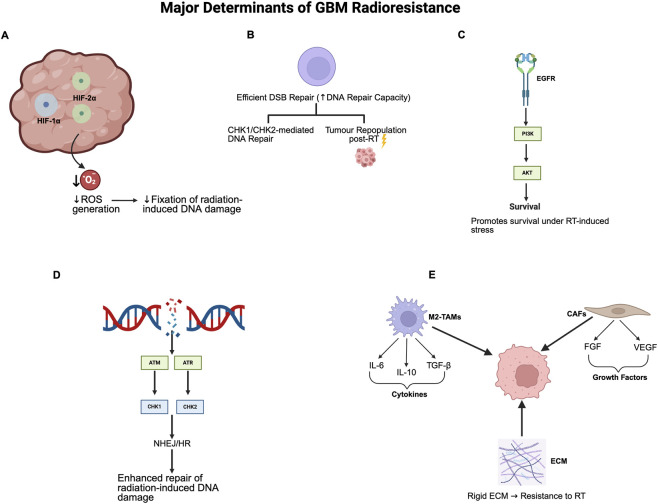
Major Determinants of GBM Radioresistance. **(A)** Hypoxic GBM cell. Hypoxia stabilises HIF-1α and HIF-2α, reducing ROS generation and limiting the fixation of radiation-induced DNA damage, thereby diminishing RT efficacy. **(B)** Glioma stem cells (GSCs). GSCs exhibit highly efficient DSB repair via CHK1/CHK2 signalling and drive post-RT tumour repopulation through enhanced DNA repair capacity. **(C)** Pro-survival signalling. EGFR–PI3K–AKT activation promotes cell survival under RT-induced stress, supporting radioresistance. **(D)** DNA damage response (DDR) activation. Radiation-induced DSBs activate ATM/ATR and downstream CHK1/CHK2, facilitating NHEJ/HR and enhancing repair of radiation-induced DNA lesions. **(E)** Tumour microenvironment. M2-polarised TAMs secrete IL-6, IL-10 and TGF-β; CAFs release FGF and VEGF; and a stiffened ECM collectively promote GBM progression, survival, and resistance to RT. Created in BioRender. Ray, H. (2026) https://BioRender.com/3ebyh2q.

Together, these factors contributing to radioresistance (Figure 1) underscore the need for radiosensitisers. Radiosensitisers enable radiotherapy to remain feasible and effective by maintaining the radiation dose, which would otherwise damage normal tissues (Wang et al., 2018). They increase the sensitivity of cells to radiation through alteration of cell factors’ activity which regulate the detrimental effects of radiation (Wang et al., 2018). Radiosensitisation operates via several mechanisms such as intracellular thiol and repair biomolecule inhibition, cytotoxic substance production and mimicking of electrophilic oxygen activity (Wang et al., 2018; Shestovskaya et al., 2023; Adams et al., 1976; Bump and Brown, 1990).

## 3 Therapeutic approaches to radiosensitisation

### 3.1 Pharmacological radiosensitisers

Targeted delivery of Mouse Double Minute 2/X Homolog (MDM2/X) inhibitors represents a promising radiosensitisation strategy, as these agents can induce targeted cell death of glioma cells and limit toxicity in normal tissues. They enhance radiosensitivity by inhibiting proteins such as MDM2, which mediates ubiquitin-dependent degradation and therefore downregulation of tumour suppressor p53 (Miles et al., 2021). Approximately 84% of GBM cases exhibit dysregulated p53-MDM2 pathway (Miles et al., 2021). The DNA damage response (DDR) pathway modulates the p53-MDM2 signalling via activated kinases such as ATM (Miles et al., 2021; Spiegelberg et al., 2018). Additionally, radiosensitisers operate by regulating cellular components of DDR pathways such as DNA damage sensors, proximal and distal signal transducers, cell cycle regulators, transcription factors, apoptosis mediators and DNA repair proteins (Miles et al., 2021). High-Z metal nanoparticles also function as radiosensitisers through physical dose amplification and subsequent biological effects on the tissue such as oxidative stress, ROS-mediated DNA damage, cell cycle and bystander effects (Choi et al., 2020; Butterworth et al., 2012).

Radiosensitisers function by exploiting the TME and signalling pathways. For instance, autophagy inhibitor bafilomycin A1 was reported to diminish the neurosphere-forming ability of GSCs and Cathepsin D inhibition was demonstrated to be positively associated with autophagic acitivity, using Small Interfering RNAs (siRNAs) has also been shown to subdue autophagy and radiosensitise GBM cells (Dalisay et al., 2024). miRNA-based approaches targeting radiation-related molecular pathways have also emerged as promising GBM radiosensitisers (B et al., 2019).

### 3.2 Natural-product-based radiosensitisers

Several natural products are under investigation to determine their potent anti-glioma effects. Particularly marine-derived compounds display several significant biological properties such as cytotoxicity, antibacterial, antifungal, antiparasitic, antiviral and antitumour properties (Liu et al., 2024). Marine-derived natural compounds have shown to have higher toxicity against GBM in comparison to TMZ, making them viable candidates for chemotherapy (Liu et al., 2024). Aberrancies in multiple signalling pathways play an essential role in the anti-glioma effects of the marine-derived natural compounds–EGFR, PI3K/AKT/mTOR and the ERK/MAPK signalling pathways. These compounds influence diverse glioma cell behaviours including cell proliferation, migration, invasion, metabolism, stemness, apoptosis and immune response (Liu et al., 2024; Khotimchenko et al., 2021).

Within the natural-product landscape, the prodiginine family–to which PG belongs–is of particular relevance. Structural congeners including undecylprodigiosin, cycloprodigiosin, metacycloprodigiosin, butylcycloheptylprodigiosin, and streptorubin B share the tripyrrole or dipyrrin pharmacophore and a common proapoptotic, DNA-interacting profile (Li et al., 2022). The clinically advanced analogue obatoclax (GX15-070) has progressed furthest, and its *in vitro* synergy with ionising radiation in oral squamous cell carcinoma provides the most clinically mature radiosensitisation precedent for the class (Sulkshane and Teni, 2016).

Direct radiation combinations have also been reported for the parent pigment and its congeners. PG enhanced gamma-radiation (6 Gy)-induced apoptosis in Caco-2 colon carcinoma cells through BCL-2/caspase-3 engagement and suppression of MAPK/TNF-α/NLRP3 inflammatory signalling (ElBakary et al., 2025), while undecylprodigiosin increased the cytotoxicity of low-dose (one to three Gy) gamma-irradiation at least fivefold in MCF-7 breast cancer cells (Arshadi et al., 2021). The latter study also illustrates an important caveat: the radiosensitising effect was radiation-dose-dependent and reversed to radioprotection at a higher dose (5 Gy), and was observed in normal dermal fibroblasts as well as tumour cells–underscoring that tumour selectivity cannot be assumed and must be established directly. Collectively, these data position PG within a chemically coherent family of radiation-interacting natural products.

## 4 Prodigiosin: biological properties and Anti-GBM mechanisms

### 4.1 Source, structure and biosynthesis

Marine bacteria are major producers of bioactive cytotoxic metabolites which have high anti-cancer potential (Lin et al., 2020). Prodigiosin (PG) is a red tripyrrole pigment, a secondary metabolite commonly secreted by *Serratia marcescens* or other Gram-negative bacteria (Williamson et al., 2005). Its biosynthetic pathway is known to involve distinct pathways for the production of monopyrrole 2-methyl-3-n-amyl-pyrrole (MAP) and the bipyrrole, 4-methoxy-2,2′-bipyrrole-5-carbaldehyde (MBC) (Pérez-Tomás et al., 2003). MAP and MBC are then coupled in the final condensation step which utilises *pig* gene cluster-encoded enzymes, such as PigB, PigD and PigE for the synthesis of MAP and PigA, PigG and PigI for the synthesis of MBC (Pérez-Tomás et al., 2003).

### 4.2 Cytosolic acidification and mitochondria-mediated apoptosis

Alterations in intracellular pH (pH_i_) within acidic organelles might trigger or regulate apoptosis. An early mitochondrial-dependent apoptotic event includes an alteration in cellular pH modulation marked by mitochondrial alkalinisation and auxiliary cytosolic acidification (Pérez-Tomás et al., 2003; Matsuyama et al., 2000). PG-induced mitochondrial acidification disrupts proton gradients and leads to loss of mitochondrial membrane potential (ΔΨm), a hallmark early event that precedes mitochondrial outer membrane permeabilisation and cytochrome c release. Subsequent to these changes, Cytochrome c is released, mediating cytosolic activation of caspases (Pérez-Tomás et al., 2003; Matsuyama et al., 2000). Part of PG’s mechanism of action is contingent on its ability to split the coupled vacuolar H^+^-ATPase (V-ATPase) by aiding the H^+^/Cl^−^ symporter and acid neutralisation in the cells, thereby inducing intracellular acidification and eventually apoptosis (Pérez-Tomás et al., 2003; Castillo-Ávila et al., 2005).

Protonophore cPrG·HCl raises lysosomal pH by hindering V-ATPase proton pump activity without affecting its ATPase function (Pérez-Tomás et al., 2003; Lee et al., 1995). Furthermore, Cl^−^ is required for collapsing the chemical gradient of H^+^ across the tonoplast, during the inhibition of vacuolar acidification by cPrGHCl (Pérez-Tomás et al., 2003; Nakayasu et al., 2000). cPrGHCl inhibits lysosomal acidification, reduces pH_i_ and induces apoptosis in human breast cancer cells which upregulate V-ATPase and maintain a higher pH_i_ compared to normal cells, suggesting that high pH_i_ is required for the preservation of the function of cancer cells which have higher sensitivity to pH changes than non-cancerous cells (Pérez-Tomás et al., 2003; Yamamoto et al., 2000a). This hypothesis is supported by other studies in human myelocytic leukaemia cells (HL-60) and in colon cancer cell lines (Yamamoto et al., 2001; Yamamoto et al., 2000b).

PG, metacycloprodigiosin and Undecylprodigiosin (UP) exhibit H^+^/Cl^−^ symport activity on liposomal membranes in order to uncouple the F- and V-ATPases, without inhibiting catalysis or membrane potential formation (Pérez-Tomás et al., 2003; Sato et al., 1998). A study reported that three pyrrole units are necessary for PGs to hinder vacuolar acidification. By utilising 2 PG derivatives anticipated to affect only one of either biological responses–murine spleen cell proliferation or activity inhibiting acidification of vacuoles, they discovered that PG’s antiproliferative effects appear to involve mechanisms distinct from vacuolar acidification inhibition (Pérez-Tomás et al., 2003; Songia et al., 1997). Therefore, the ability of PG to disrupt proton gradients and induce cytosolic acidification may sensitise GBM cells to radiation therapy-induced apoptosis.

### 4.3 ER stress and autophagy-associated cell death

PG induces apoptosis in U87MG and GBM8401 cell lines by significantly increasing caspase-3 activity in a dose-dependent manner (Cheng et al., 2018). Heightened levels of cleaved-PARP was also found in PG-treated GBM cells (Cheng et al., 2018). PG strongly colocalises with ER marker calnexin, although moderately correlated with mitochondrial marker cytochrome c and very weakly correlated with ribosomal marker S6 in the GBM8401 cells’ cytosol (Cheng et al., 2018). In PG-treated GBM cells, ER stress marker sXBP1 mRNA, stress-induced autophagy regulator Binding immunoglobulin protein (BiP)/GRP78 and ER stress-induced proapoptotic factor CHOP, were upregulated (Cheng et al., 2018). LC3 (autophagy-detecting marker) puncta formation was also observed in the PG-treated GBM8401 cells, although LC3 protein was not colocalised with PG in the cytosol (Cheng et al., 2018). Following PG treatment, the LC3-II/LC3-I ratio and phospho-JNK levels both increased, consistent with JNK activation as a driver of autophagic cell death (Cheng et al., 2018).

PG reduced autophagy and apoptosis initiators AKT and mTOR and S6 phosphorylation in a treatment time-dependent manner in GBM8401 and U87MG cells within 24 h (Cheng et al., 2018). It was also observed that PG-treated cells showed decreased levels of p62 whose inhibition caused autophagic apoptosis and increased autophagic apoptotic effector Bax and LC3-II/LC3-I ratio (Cheng et al., 2018). Autophagy inhibitor 3-MA prevented PG-induced LC3 puncta accumulation and significantly increased PG-induced cytotoxicity (Cheng et al., 2018). Autophagy inhibitors Wortmannin, Bafilomycin A1 and 3-MA did not induce apoptosis in GBM8401 cells revealing that PG partly induces autophagic cell death in GBM cells (Cheng et al., 2018).

### 4.4 PG-induced cell cycle arrest

Research has also shown that PG induces inhibition of cell proliferation in the LN229, U251 and A172 GBM cell lines. PG also induced cell cycle arrest by inhibiting Akt phosphorylation while simultaneously aiding p21 and p53 expression (Zhao et al., 2022). However, this effect was reverted by increasing phospho-Akt levels. PG blocks the cell cycle via the KIAA1524/PP2A signalling pathway (Zhao et al., 2022). The PP2A inhibitor KIAA1524 mRNA levels were significantly reduced in all the three PG-treated GBM cell lines (Zhao et al., 2022). The role of this pathway in the antitumour activity of PG was confirmed by upregulating the expression of KIAA1524 in the LN229 cell line via lentiviral transduction (Zhao et al., 2022). The alteration in PG-induced protein expression was reversed by exogenous expression of KIAA1524 (Zhao et al., 2022).

### 4.5 DNA damage induction

PG’s planar structure allows intercalation to facilitate DNA binding while the methoxy and nitrogen rings bind DNA by providing hydrogen-binding sites (Pérez-Tomás et al., 2003). At physiological pH, PG mediates electrostatic interaction with the negatively charged phosphate groups of the DNA helix, owing to its cationic nature. PG is a DNA interacting agent favouring minor-groove AT sites (Melvin et al., 1999). Furthermore, electron-rich PG undergoes oxidation thereby facilitating oxidative double strand (ds) DNA cleavage promoted by the reductive activation of Cu (II) (Melvin et al., 2000). Copper is a crucial trace element distributed in all cellular organelles and nucleus and is found in high amounts in cancerous cells–in dry non-cancerous breast tissue, its mean concentration is 1.47 ppm, rising to 5.12 ppm in cancerous tissue (Sarkar, 2000; Theophanides and Anastassopoulou, 2002). A study suggested that any damage under these conditions would be substantial and should be fatal to the cells, and suggested a correlation between nuclease activity and PG’s cytotoxicity (Melvin et al., 2001).

The redox properties of pyrromethene are affected by PG’s A-pyrrole ring (Pérez-Tomás et al., 2003). The intact pyrrolylpyrromethene chromophore of PGs is necessary for the more lethal copper-aided dsDNA cleavage agent whereas the bipyrrole moiety aids ssDNA cleavage (Melvin et al., 2001; Melvin et al., 2002). DNA damage causes cells to respond by the activation of an intricate DNA-damage response pathway involving cell cycle arrest, DNA repair and under certain circumstances, the induction of apoptosis (Fürstner and Grabowski, 2001; Khanna and Jackson, 2001). Due to their ability of DNA binding, PGs are able to disrupt its replication triggering apoptosis, as implied earlier, and therefore are prospective anticancer drug candidates (Pérez-Tomás et al., 2003).

### 4.6 Anti-glioma effects: preclinical evidence

Preclinical evidence suggests that PG induces ER stress and autophagy in U87MG GBM cells, however its role as a radiosensitiser remains underexplored. A study showed that PG induces apoptosis and morphological changes in human GBM cells, and significantly found to reduce cell viability in a dose-dependent manner in the U87MG, GBM8401 and SVGp12 cell lines (Cheng et al., 2018). Microscopic observation also revealed the formation of large swollen autophagic vacuoles in U87MG and GBM8401 cells (Cheng et al., 2018). 0.1 μM PG reduced growth of neurospheres compared to 200 μM TMZ producing the same effect, suggesting that PG depletes the self-regenerating GBM population at a concentration much lower than that of TMZ (Cheng et al., 2018). The effects of PG were also demonstrated *in vivo* in the LN229 mouse xenograft model and PG treatment reduced the protein level of KIAA1524 in the *in vivo* tumour samples thereby suggesting that PG-induced attenuation of KIAA1524 can prevent tumour development (Zhao et al., 2022).

### 4.7 Photosensitising properties

Beyond its activity in the dark, PG and synthetic prodigiosenes also display light-dependent cytotoxicity. Three prodigiosenes–a synthetic prodigiosin analogue and two estrogen-receptor-targeting conjugates–photosensitise the generation of singlet oxygen and exert photo-induced cytotoxicity against breast cancer cell lines, indicating photodynamic-therapeutic potential for the tripyrrole class (Savoie et al., 2018). Alkylated prodigiosenes and their Cu(II) complexes show comparable photoinduced anticancer activity, with the Cu(II) complex of the α-unsubstituted prodigiosene proving most active and also engaging DNA (Kettenmann et al., 2022). These findings establish that prodiginines can act as visible-light photosensitisers. It is important to distinguish this mechanism from radiosensitisation. Photodynamic ROS generation arises from excited-state energy transfer to molecular oxygen under visible-light irradiation, whereas ionising radiation generates ROS through direct ionisation and radiolysis of water. The two converge on a ROS-mediated cytotoxic endpoint but differ fundamentally in their initiating physics, and conflating them would overstate the radiosensitisation case. The photosensitisation literature is therefore presented here as evidence of PG’s broader oxidative-sensitising profile, not as a direct mechanistic precedent for ionising-radiation radiosensitisation.

## 5 PG pleiotropic signalling mechanisms

### 5.1 Modulation of MAPK signalling pathways

PGs have been shown to influence MAPK signalling cascades in various ways. These cascades include stress-activated protein kinase (SAPK)/c-jun N-terminal kinase (JNK) and p38-MAPK, induced by cytokines, stress responses and a mediator of differentiation and apoptosis and the ERKs which are generally associated with growth factors and proliferation (Pérez-Tomás et al., 2003). Moreover, PGs either activate one or both of the SAPK/JNK and p38-MAPK pathways, thereby inducing cell death, although PG triggered p38 phosphorylation but not of JNK-MAPK (Montaner and Pérez-Tomás, 2002). Stress-activated p38 and JNK signalling contributes to radiation-induced apoptosis, an axis GBM cells dampen as part of their radioresistant phenotype–so PG’s preferential activation of p38-MAPK may reinforce the pro-death stress signalling that irradiation engages rather than opposing it.

### 5.2 PI3K–Akt–mTOR axis

PG has been demonstrated to suppress the PI3K–Akt–mTOR axis across malignancies beyond the GBM-specific Akt, mTOR and S6 inhibition. PG induces autophagic cell death in doxorubicin-sensitive and -resistant lung adenocarcinoma cells through inhibition of PI3K–p85/Akt/mTOR signalling and through a parallel Beclin-1/PI3K-Class III-independent pathway (Chiu et al., 2018). Combination of PG with the HSP90α inhibitor PU-H71 in MDA-MB-231 triple-negative breast cancer cells reduced mTOR, EGFR and VEGF expression and elevated caspase-3, -8 and -9 activation (Anwar et al., 2020). Since Akt suppression impairs γ-H2AX foci resolution in irradiated GBM cells, PG-mediated Akt inhibition across these tumour types reinforces the same radiosensitising mechanism in GBM.

### 5.3 p53/p73 reactivation in p53-deficient tumours

PG and its structural analogue compound R restore p53 pathway activity in p53-deficient and p53-mutant colon cancer cells, inducing p53 target genes (p21, PUMA, NOXA, BAX), cell-cycle arrest and apoptosis at nanomolar concentrations and without cytotoxicity to normal fibroblasts (Hong et al., 2014)

PG drives c-Jun-mediated upregulation of p73 and downregulation of the oncogenic ΔNp73 isoform in 5-FU-resistant Aldefluor^+^ colorectal cancer stem cells, suppressing self-renewal *in vitro* and limiting tumourigenicity *in vivo* (Prabhu et al., 2016). PG-induced p53 accumulation and survivin reduction were also demonstrated in T-cell acute lymphoblastic leukaemia Molt-4 cells (Sam and Pourpak, 2018). TP53 is mutated in roughly 25%–30% of primary GBM, and defective p53-dependent apoptotic signalling has been mechanistically linked to the intrinsic radioresistance of GBM cells (Zhang Y. et al., 2018; Haas-Kogan et al., 1996). PG-mediated p53/p73 reactivation may therefore restore radiation sensitivity in GBM tumours that have lost wild-type p53 function.

### 5.4 BCL-2 family interactions

Beyond the direct BH3-domain engagement detailed in Section 6.3 (Hosseini et al., 2013), PG also lowers BCL-2 family activity at the transcriptional level. PG-mediated CHOP induction also transcriptionally suppresses BCL-2 in breast carcinoma cells through engagement of the IRE1–JNK and PERK–eIF2α arms of the unfolded protein response (Pan et al., 2012). GBM cells frequently overexpress BCL-2 and MCL-1, which sequester pro-apoptotic effectors and elevate the threshold for radiation-induced apoptosis (Wolfsperger et al., 2016). PG’s combined direct binding of BCL-2 family members and transcriptional suppression of BCL-2 may therefore lower the apoptotic threshold for radiation-induced mitochondrial stress in GBM.

### 5.5 Survivin (BIRC5) suppression

PG suppresses survivin transcription and protein expression across multiple cancer types. In HepG2 hepatocellular carcinoma cells, 600 nM PG reduced survivin mRNA by 88.5% and increased caspase-3 activation by 330% (Yenkejeh et al., 2016). PG and the structural analogues cyclononylprodigiosin and nonylprodigiosin downregulate survivin and increase γ-H2AX-positive DNA damage and caspase-3 cleavage in melanoma cells (Branco et al., 2020). PG produced concentration-dependent survivin reductions in T-ALL Molt-4 cells alongside p53 accumulation and caspase-3 activation (Sam and Pourpak, 2018). Survivin is overexpressed in GBM and correlates with poor survival and radioresistance, and is also implicated in homologous-recombination repair of radiation-induced DSBs (Chakravarti et al., 2004; Véquaud et al., 2015). PG-mediated survivin suppression therefore aligns with the requirements of a multi-mechanistic GBM radiosensitiser.

### 5.6 GSK-3β activation and AKT–GSK-3β cross-talk

PG triggers Akt dephosphorylation and consequent activation of GSK-3β in MCF-7 breast cancer cells, driving induction of the proapoptotic gene NAG-1 and upregulation of death receptors DR4 and DR5 (Soto-Cerrato et al., 2007). Pharmacological inhibition of GSK-3β rescues cells from PG-induced apoptosis (Soto-Cerrato et al., 2007). Akt phosphorylates and inhibits GSK-3β under normal growth conditions; PG-mediated Akt suppression therefore releases GSK-3β to engage extrinsic and intrinsic apoptotic outputs. Constitutive Akt activation is a hallmark of GBM radioresistance, and PG-mediated relief of GSK-3β inhibition may amplify radiation-induced cell death by activating death receptor signalling.

### 5.7 Inhibitor of apoptosis (IAP) family

PG downregulates XIAP, cIAP-1 and cIAP-2 in choriocarcinoma JEG3 and prostate cancer PC3 cells *in vitro* and in xenograft models, with corresponding activation of caspase-9 and caspase-3 and elevated Bax/Bcl-2 ratios (Li et al., 2018). IAP-family suppression aligns mechanistically with the survivin data in 5.5 and contributes to the broad apoptotic-priming profile of PG. XIAP and the cIAPs directly restrain the caspase cascade that executes radiation-induced apoptosis, so PG-mediated depletion of these inhibitors may lower the barrier to apoptosis in irradiated GBM cells and complement radiotherapy-induced mitochondrial injury.

### 5.8 Pathway convergence on radiation response

**FIGURE 2:**
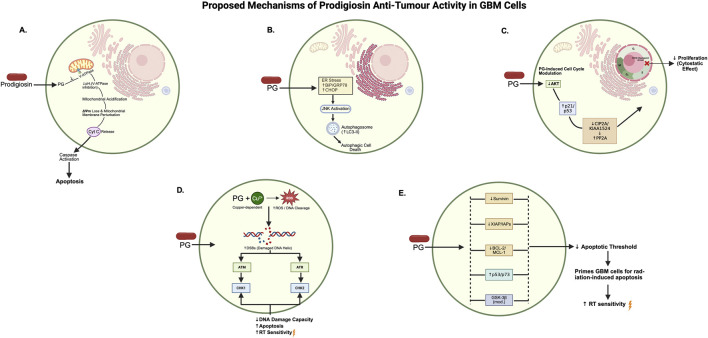
Proposed Mechanisms of Prodigiosin (PG) Anti-Tumour Activity in GBM Cells. **(A)** PG inhibits V-ATPase-dependent proton transport, inducing cytosolic acidification, mitochondrial acidification, ΔΨm loss, membrane perturbation, cytochrome c release, caspase activation and apoptosis. **(B)** PG induces ER stress (↑BiP/GRP78, ↑CHOP, ↑sXBP1), activates JNK, promotes LC3-II accumulation and autophagosome formation, culminating in autophagy-associated cell death. **(C)** PG suppresses AKT phosphorylation and increases p21/p53 expression, while downregulating KIAA1524/CIP2A to activate PP2A, resulting in G1/S arrest and reduced proliferation. **(D)** PG, in the presence of Cu^2+^, enhances ROS-mediated DNA cleavage and increases DSB burden. Concurrent inhibition of AKT–CHK signalling decreases DDR capacity, increasing apoptosis and radiosensitivity. **(E)** PG pleiotropically tips the apoptotic balance in GBM–suppressing the anti-apoptotic effectors survivin, XIAP and BCL-2/MCL-1 while restoring pro-apoptotic p53/p73 and modulating GSK-3β. Convergence of these effects lowers the apoptotic threshold, priming GBM cells for radiation-induced apoptosis. Created in BioRender. Ray, H. (2026) https://BioRender.com/d0tejzm.

Together, these data indicate that PG simultaneously suppresses multiple parallel pro-survival signalling axes–PI3K–Akt–mTOR, survivin/IAPs and BCL-2/MCL-1 – rather than acting on a single target (Figure 2). Several of these pathways (Akt, survivin, BCL-2 family) are independently implicated in DSB repair efficiency and post-irradiation checkpoint recovery. PG can also reactivate p53/p73-dependent transcription in p53-deficient cells, offering a route to restoring radiation sensitivity in TP53-mutant GBM. The convergence of these effects on radioresistance-relevant nodes supports the rationale for PG as a multi-mechanistic GBM radiosensitiser, and identifies γ-H2AX persistence, survivin and MCL-1 levels and p53/p73 reporter activity as priority biomarkers for the experimental programme outlined in Section 8.

## 6 Direct molecular targets and target engagement

Though PG’s pleiotropic signalling effects are extensively documented, identification of its direct molecular targets has trailed behind its phenotypic characterisation. PG exhibits cytotoxic potency in the low-nanomolar to low-micromolar range across diverse cancer cell lines, but the binding events underlying this activity have only recently been resolved using affinity-based methods. Several direct or functionally engaged targets have now been demonstrated, providing molecular anchors for the radiosensitisation hypothesis advanced in this review.

### 6.1 GRASP55: the first direct target identified by thermal proteome profiling

A study has identified the Golgi reassembly stacking protein GRASP55 (GORASP2) as a direct target of PG using mass spectrometry-based thermal proteome profiling (TPP) in HeLa cells (Berning et al., 2023). TPP exploits the thermal stabilisation that intracellular proteins acquire on small-molecule binding, extending the cellular thermal shift assay (CETSA) to a proteome-wide scale. In the temperature-range arm (TPP-TR), differential melting and abundance data were obtained for 2,480 proteins meeting the analysis quality criteria, and GRASP55 emerged as the most statistically significant thermally stabilised protein, with a mean melting point shift of ΔTm ≈2.1 °C (Berning et al., 2023). Stabilisation occurred at a steady GRASP55 abundance, indicating a binding rather than an expression effect (Berning et al., 2023). CRISPR/Cas9-mediated GRASP55 knockout partially reduced PG cytotoxicity, indicating that GRASP55 contributes mechanistically to PG action (Berning et al., 2023).

The functional consequences converge on the autophagy–lysosome axis. PG treatment severely disrupts Golgi morphology, decreases cathepsin activity and blocks autophagic flux, while promoting colocalisation of the autophagosomal marker LC3 with the lysosomal marker LAMP1 and driving accumulation of autophagosomes at GRASP55-positive structures (Berning et al., 2023). GRASP55 bridges autophagosomes and lysosomes by binding LC3-II on the autophagosome and LAMP2 on the lysosome, functioning as a membrane tether required for autophagosome–lysosome fusion (Zhang X. et al., 2018). The GRASP55 finding therefore unifies several previously fragmented observations–PG’s effects on autophagic flux, lysosomal function and Golgi integrity–around a defined molecular target. GBM cells display constitutively elevated, cytoprotective autophagy in response to ionising radiation (Aiyappa-Maudsley et al., 2022); targeting GRASP55-dependent autophagy may convert this protective response into autophagic cell death in irradiated GBM.

### 6.2 V-ATPase: functional engagement of the lysosomal proton pump

PG and several prodiginine analogues act as H+/Cl-symporters that uncouple vacuolar-type ATPase (V-ATPase) activity, dissipating the proton gradient across endolysosomal and Golgi membranes (Nakayasu et al., 2000; Ohkuma et al., 1998). This activity requires neither catalytic inhibition of V-ATPase nor collapse of the membrane potential, and depends on the intact tripyrrole core, since two-pyrrole derivatives lose vacuolar-acidification activity (Pérez-Tomás et al., 2003). Direct binding studies against purified V-ATPase subunits have not been reported, so the interaction is best described as functional rather than biophysically characterised engagement. It nonetheless underlies PG’s effects on cytosolic acidification, lysosomal cathepsin inhibition and autophagic-flux blockade, and may operate in parallel with GRASP55 binding within the endomembrane system. In the radiosensitisation context, this proton-gradient disruption reinforces the mitochondrial and lysosomal stress that PG is proposed to add to radiation-induced injury in GBM.

### 6.3 BCL-2 family proteins: BH3-domain binding and MCL-1 antagonism

PG and obatoclax–a clinically advanced prodiginine analogue–bind the BH3-binding groove of anti-apoptotic BCL-2 family members, with molecular modelling indicating the highest predicted affinity for MCL-1 (Hosseini et al., 2013). PG displaces BAK from preformed MCL-1/BAK complexes in melanoma cells, releasing BAK to drive mitochondrial outer membrane permeabilisation (Hosseini et al., 2013). Obatoclax antagonises MCL-1 and overcomes MCL-1-mediated resistance to apoptosis, inhibiting BH3-peptide binding across all six pro-survival family members with reported fluorescence-polarisation Ki values of approximately 1–7 µM (Véquaud et al., 2015). Its hydrophobicity (calculated logP >4) renders absolute affinities measured in aqueous biochemical assays unreliable, but cellular target engagement is unambiguous (Nguyen et al., 2007). Critically for this review, obatoclax synergises with ionising radiation *in vitro* in oral squamous cell carcinoma cells, producing significant clonogenic inhibition relative to either treatment alone–the most direct radiosensitisation precedent currently available for the prodiginine class (Sulkshane and Teni, 2016). GBM cells frequently overexpress MCL-1 and BCL-2, which sequester pro-apoptotic effectors and raise the apoptotic threshold for radiation-induced cell death (Wolfsperger et al., 2016). PG’s direct BCL-2 family engagement therefore provides a mechanistic template for the GBM radiosensitisation hypothesis.

### 6.4 mTOR: dual mTORC1/mTORC2 inhibition

PG and obatoclax directly engage mTOR in melanoma cells, inhibiting both mTORC1 and mTORC2 (Espona-Fiedler et al., 2012). Direct binding was demonstrated by surface plasmon resonance and the binding mode characterised by *in silico* modelling, with PG producing its strongest effect on mTORC2 (89% activity inhibition) and abolishing AKT phosphorylation at Ser473 without affecting Thr308 (Espona-Fiedler et al., 2012). This direct, dual-complex engagement is mechanistically distinct from the indirect mTOR suppression that follows PG-mediated Akt inhibition. PI3K/mTOR-pathway signalling sustains DNA double-strand break repair and post-irradiation survival in GBM–dual PI3K/mTOR inhibition attenuates DSB repair through DNA-PKcs and ATM and radiosensitises orthotopic glioblastoma models–so inhibition of this axis is functionally consequential for radiosensitisation (Gil del Alcazar et al., 2014).

### 6.5 Cu(II): a non-protein molecular partner

PG coordinates Cu(II) to generate a redox-active complex that catalyses oxidative double-strand DNA cleavage (Fürstner and Grabowski, 2001; Khanna and Jackson, 2001). Cleavage proceeds through reductive activation of Cu(II) to Cu(I), driven by oxidation of the electron-rich PG molecule, with the bipyrrole moiety central to this oxidative nuclease activity (Khanna and Jackson, 2001). Because cancer tissues exhibit elevated copper relative to non-malignant tissue, PG-mediated Cu(II)-dependent DNA damage is predicted to be tumour-selective. This profile aligns directly with the pharmacological output desired of a radiosensitiser–additional ROS-mediated DNA damage superimposed on radiation-induced lesions–and identifies Cu(II) as a non-protein molecular partner of mechanistic relevance comparable to the protein targets above.

### 6.6 Methodological landscape and remaining gaps

**FIGURE 3:**
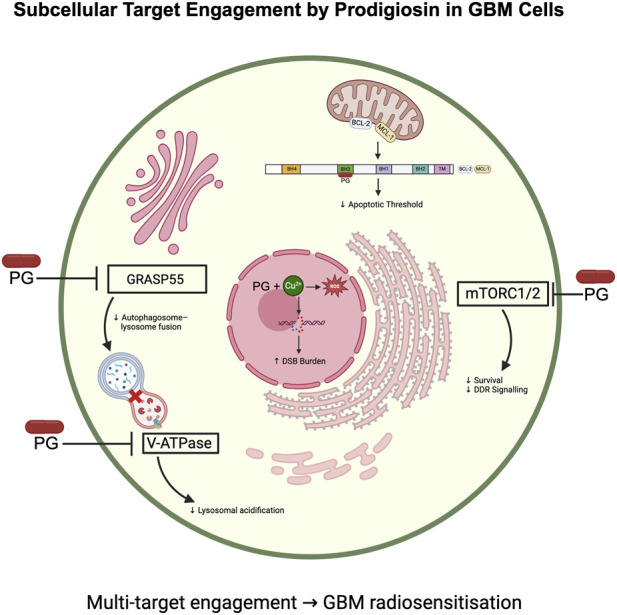
Direct molecular targets of prodigiosin (PG) mapped to their subcellular sites in GBM cells. Created in BioRender. Ray, H. (2026) https://BioRender.com/wqcgjrp.

The affinity-based methods now standard for natural-product target identification include thermal proteome profiling and CETSA, surface plasmon resonance, isothermal titration calorimetry, photoaffinity-labelling chemoproteomics, and DARTS or pulse-proteolysis. Of these, only TPP/CETSA has been comprehensively applied to PG to date, and PG’s hydrophobicity (logP > 4) constrains the choice of aqueous biophysical assays. Together, the available data indicate that PG engages a small set of high-affinity protein targets–GRASP55, the BCL-2 family and mTOR–distributed across distinct subcellular compartments (Figure 3), supplemented by metal-coordination chemistry (Cu(II)-dependent DNA cleavage) and a functional interaction with V-ATPase. Each target is independently implicated in the cancer-cell radiation response, providing convergent mechanistic support for the radiosensitisation hypothesis. Photoaffinity probes, surface plasmon resonance with detergent-solubilised targets, and protein-fragment complementation assays could extend this picture and are identified as priorities in Section 8.

PG inhibits GRASP55 at the Golgi, impairing autophagosome–lysosome fusion, and inhibits lysosomal V-ATPase, reducing lysosomal acidification–together suppressing the cytoprotective autophagy that GBM cells mount against irradiation. PG binds the BH3 groove of the anti-apoptotic regulators BCL-2 and MCL-1, lowering the apoptotic threshold, and inhibits mTORC1/2, attenuating pro-survival and DNA-damage-response signalling. Within the nucleus, the PG–Cu(II) complex generates reactive oxygen species that drive oxidative double-strand breaks, increasing the DSB burden. Convergence of these engagements across distinct subcellular compartments supports a multi-mechanistic basis for PG-mediated GBM radiosensitisation.

## 7 Rationale for prodigiosin as a GBM radiosensitiser

### 7.1 Mechanistic synergy with radiotherapy

**FIGURE 4:**
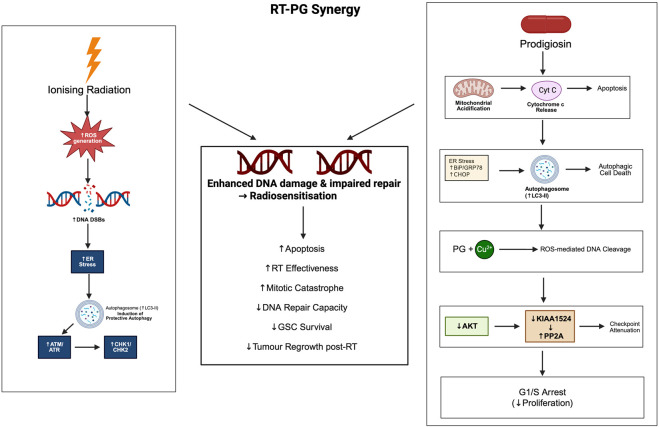
Conceptual Framework of PG–Radiotherapy (RT) Synergy in GBM. Left panel: Ionising radiation increases ROS generation and induces DNA DSBs, ER stress, autophagy induction and DDR activation (ATM/ATR → CHK1/CHK2), promoting protective repair responses. Right panel: PG induces mitochondrial acidification, cytochrome c-mediated apoptosis, ER stress-associated autophagy, Cu^2+^-dependent ROS/DNA damage, KIAA1524 suppression (↑PP2A), inhibition of AKT signalling and G1/S arrest. Central panel: Convergence of PG-mediated DNA damage, mitochondrial dysfunction, checkpoint attenuation and impaired DDR enhances radiation-induced cytotoxicity. This synergy results in increased apoptosis, mitotic catastrophe, reduced DNA repair capacity, suppression of GSC survival and diminished tumour regrowth post-RT. Created in BioRender. Ray, H. (2026) https://BioRender.com/3hu7t3b.

Ionising radiation mainly induces tumour apoptosis through DNA DSBs and mitochondrial-dependent cell death, but GBM cells often evade this damage by efficiently activating DNA repair and survival signalling pathways. PG causes oxidative DNA damage and disrupts the repair processes by aiding DSB formation dependent on copper and by inhibiting Akt signalling, a fundamental modulator of GBM DNA damage response. Since hyperactivation of Akt accelerates γ-H2AX foci resolution following exposure to radiation in resistant tumourigenic cells, PG-mediated decrease in Akt phosphorylation may impair DNA repair, thereby potentiating radiation-induced DNA damage. This Akt suppression is now traceable to direct target engagement–PG binds mTOR and inhibits mTORC2, abolishing AKT phosphorylation at Ser473 (Espona-Fiedler et al., 2012) – and dual PI3K/mTOR inhibition attenuates DSB repair through DNA-PKcs and ATM to radiosensitise orthotopic GBM models (Gil del Alcazar et al., 2014). Together, these mechanisms suggest that PG could synergise with radiotherapy by overpowering the DNA repair capacity of GBM cells and promoting apoptosis in response to radiation-induced stress (Figure 4).

IR also increases ROS production under normoxic conditions leading to DNA damage, but hypoxia as found in GBM TME causes a decline in ROS production. PG lowers cytosolic pH via uncoupling of V-ATPase, leading to mitochondrial acidification, release of cytochrome c and caspase activation and finally apoptosis. Therefore, PG-induced mitochondrial stress and cell death may amplify ROS production, intensifying radiation-induced ROS production which would lead to DNA damage. Together, these mechanisms implicate that PG may synergise with radiotherapy by intensifying mitochondrial stress and cell death as a result of radiation-induced ROS production.

Irradiation of GBM cells leads to protective autophagy by inhibition of Akt/mTOR activity. Evidence has also demonstrated that PG partly plays a role in inducing ER stress and therefore autophagic cell death in GBM cells by JNK activation. This block now has a defined molecular basis–PG directly targets GRASP55, the Golgi-resident tether required for autophagosome–lysosome fusion, arresting autophagic flux at a specific node rather than through diffuse stress signalling alone (Berning et al., 2023; Zhang X. et al., 2018). Hence, PG-mediated ER stress and autophagy may convert radiation-induced cytoprotective autophagy into autophagic cell death. Together, these mechanisms indicate that PG may overcome radiation-induced cytoprotective autophagy.

Radiotherapy preferentially eliminates differentiated GBM cells while sparing glioma stem cells (GSCs), which possess enhanced DNA repair capacity and activation of PI3K-Akt and checkpoint signalling pathways. This selective survival results in GSC enrichment following irradiation and contributes significantly to tumour recurrence. PG markedly reduces neurosphere formation in GBM models, indicating a direct effect on the self-renewing tumour cell population. By depleting GSCs, PG could reduce the reservoir of radioresistant cells that drive relapse after treatment, thereby complementing radiotherapy-induced cytotoxicity and limiting recurrence.

Ionising radiation is most effective during the G2/M cell cycle phase, however GBM cells frequently evade damage by arresting in phases less sensitive to irradiation. PG induces cell-cycle arrest in GBM cell lines by inhibiting Akt phosphorylation and upregulating p21 and p53, thereby disrupting proliferative checkpoint control. Because Akt signalling contributes to radiation-induced checkpoint recovery, PG-mediated inhibition of this pathway may prevent GBM cells from recovering from radiation-induced checkpoint arrest and escaping mitotic catastrophe following irradiation. By positioning tumour cells in more radiosensitive phases and limiting their ability to repair before division, PG may augment the cytotoxic impact of radiotherapy and limit treatment resistance.

Radiation-induced apoptosis in GBM proceeds largely through the mitochondrial pathway, yet GBM cells raise the threshold for this death by overexpressing the anti-apoptotic proteins BCL-2 and MCL-1, which sequester pro-apoptotic effectors and blunt the response to radiation-induced mitochondrial stress (Wolfsperger et al., 2016). PG binds the BH3-binding groove of these proteins–with the highest predicted affinity for MCL-1 – and displaces BAK from MCL-1/BAK complexes to drive mitochondrial outer membrane permeabilisation (Hosseini et al., 2013), while CHOP induction transcriptionally suppresses BCL-2 (Pan et al., 2012). Critically, obatoclax, a clinically advanced prodiginine analogue, synergises with ionising radiation *in vitro* to produce significant clonogenic inhibition relative to either treatment alone–the most direct radiosensitisation precedent currently available for the prodiginine class (Sulkshane and Teni, 2016). Together, these mechanisms suggest that PG could synergise with radiotherapy by removing the anti-apoptotic brake that allows GBM cells to survive radiation-induced mitochondrial injury.

### 7.2 Convergence and tumour selectivity

The mechanisms above converge on a single property: PG does not act through one target but engages a distributed set–GRASP55, the BCL-2/MCL-1 family, mTOR, V-ATPase and, as a non-protein partner, Cu(II) – spread across the Golgi, mitochondria, lysosome and nucleus (Berning et al., 2023; Hosseini et al., 2013; Espona-Fiedler et al., 2012; Ohkuma et al., 1998; Fürstner and Grabowski, 2001). Each node is independently implicated in the GBM radiation response, so their simultaneous engagement predicts additive or supra-additive radiosensitisation rather than a single point of leverage that resistance could bypass.

This multi-target profile also frames the question of tumour selectivity. PG-mediated Cu(II)-dependent DNA cleavage is predicted to be tumour-selective because malignant tissue carries elevated copper relative to normal brain (Fürstner and Grabowski, 2001; Khanna and Jackson, 2001); GBM cells depend on cytoprotective autophagy and overexpress MCL-1 and survivin to a degree that normal glia do not (Wolfsperger et al., 2016); and PG restores p53/p73-dependent apoptosis specifically in the TP53-mutant cells that comprise a substantial fraction of GBM (Hong et al., 2014; Zhang Y. et al., 2018). Each dependency is more pronounced in the tumour than in surrounding parenchyma, indicating a therapeutic window in which PG augments radiation-induced injury in GBM while sparing normal brain–the central translational question addressed in Section 8.

## 8 Future directions

### 8.1 *In vitro* validation of PG-radiation synergy

Although PG demonstrates potent cytotoxic and pro-apoptotic activity in GBM cells, its interaction with ionising radiation has not been experimentally defined. Future work should evaluate whether PG enhances radiation-induced DNA damage, mitochondrial dysfunction and cytotoxicity in conventional GBM cell lines and stem-like neurosphere models. These studies should quantify DSB burden, γ-H2AX persistence, ROS generation, clonogenic survival and autophagy dynamics (LC3 conversion, p62 turnover, and autophagic flux), to identify whether the mechanistic predictions proposed in this review translate into measurable radiosensitisation. Analogous comparison with benign astrocytes or neuronal cell populations will be fundamental for determining whether PG selectively augments the radiosensitivity of tumour cells while sparing normal brain tissue, a critical prerequisite for clinical translation.

### 8.2 PG-radiotherapy combination studies in GBM mouse models

To assess whether PG is suitable for functioning as a radiosensitiser in the complex TME, its effects must be studied in intracranial GBM *in-vivo* models using clinically prevalent irradiation regimens. Orthotopic xenografts originating from U87MG, or preferentially, patient-derived GSCs provide a platform to evaluate the combination of PG and fractionated or hypofractionated cranial irradiation, akin to existing GBM radiosensitiser research that has demonstrated improved patient outcomes such as tumour control and survival optimised irradiation regimens. Endpoints should include serial bioluminescent imaging or Kaplan-Meier survival curves, MRI-based tumour burden, γ-H2AX and cleaved caspase-3 staining, and diminution of stem-like populations *in vivo*, to determine whether PG increases radiation-induced DNA damage, apoptosis and GSC eradication. Since orthotopic implantation also allows evaluation of normal brain toxicity and blood-brain barrier (BBB) penetration, these models are appropriate for defining a therapeutic potential of PG, to compare systemic and localised delivery strategies, and to establish whether the mechanistic synergy predicted from *in vitro* and signalling data produces a meaningful improvement in therapeutic ratio *in vivo*.

### 8.3 Strategies to enhance PG delivery and BBB penetration

A major obstacle in clinical translation of PG as a radiosensitiser is its capability to reach clinically significant intracranial concentrations. Analogous to several hydrophobic natural products, PG exhibits restricted aqueous solubility and undetermined BBB permeability, which may limit its distribution to intracranial tumours. Delivery strategies thereby represent an essential component of future development. Nanoparticle-based formulations such as lipid nanoparticles, PEGylated carriers and polymeric micelles have been successfully utilised to improve CNS delivery of structurally related small molecules and enhance radiosensitiser bioavailability in orthotopic GBM models. Convection-enhanced delivery (CED) is also a convincing approach, enabling direct intraparenchymal PG infusion to attain high locoregional concentrations while diminishing systemic toxicity. Alternative routes including ligand-targeted nanoparticles, focused ultrasound-mediated BBB opening or transferrin-induced transport could further enhance PG accumulation within tumourigenic tissue. Testing these strategies in conjunction with cranial irradiation will be crucial to decide whether PG can successfully achieve BBB permeability, disseminate within diffusely infiltrative tumour borders, and maintain adequate levels to augment RT effects.

PG’s systemic disposition is equally undefined. No absorption, distribution, metabolism or excretion data have been reported for PG *in vivo*, and its high lipophilicity (logP > 4) predicts extensive plasma-protein binding and rapid hepatic clearance–properties that would constrain the free intracranial concentrations available to interact with radiation (Nguyen et al., 2007). Defining PG’s plasma half-life, tissue partitioning and CNS exposure is therefore a prerequisite rather than an afterthought: radiosensitisers depend on adequate drug exposure coinciding with the narrow window of radiation-induced DNA damage, so the pharmacokinetic profile will directly determine feasible dosing schedules and the sequencing strategies considered in Section 8.7.

### 8.4 Toxicity, selectivity and therapeutic index optimisation

A fundamental necessity for PG’s suitability as a radiosensitiser is characterising its therapeutic index within the CNS. Despite PG’s ability to display selective cytotoxicity against malignant cells *in vitro*, its effects on normal neuronal, glial and endothelial populations are poorly defined. Future studies should evaluate PG toxicity in normal astrocytes, NSCs, oligodendrocytes and microglia, both individually and coupled with RT, to determine whether radiosensitisation is tumour-selective or risks aggravating radiation-mediated CNS injury. *In vivo* dose-finding studies in orthotopic GBM models will be needed to specify maximum tolerable dosages, neurological side-effects and potential off-target organ toxicity. Accomplishing a therapeutic window in which PG augments radiation-induced damage in tumours without affecting normal brain tissue is crucial for making PG clinically feasible.

### 8.5 Structural modification and drug formulation for radiosensitisation

PG’s chemical structure forms the basis of its bioactivity, though also contributes to its hydrophobicity, stability profile and uncertain BBB penetration. Therapeutic chemistry optimisation may therefore enhance its radiosensitisation potential. Structural alterations to the tripyrrole core such as tuning the C6 methoxy group, adjusting substituent bulk to alter lipophilicity, or creating prodrug derivatives, could increase BBB permeability, decrease systemic cytotoxicity or enhance preferential accumulation in tumourigenic tissues. Similar advances in formulation aspects, including nanoemulsions, liposomal encapsulation, polymeric carriers or pH-responsive nanoparticles, may improve selective delivery to hypoxic, acidic GBM regions. Designing PG analogues or formulations suitable for radiosensitisation could potentiate its mechanistic synergy with radiation while improving pharmacokinetics and therapeutic range.

### 8.6 Genomic and metabolic profiling to identify responders

Since GBM demonstrates profound intertumoural heterogeneity, PG-based radiosensitisation might prove to be the most effective in specific molecular subtypes. Merging genomic, transcriptomic and metabolic profiling into preclinical testing will help characterise biomarkers anticipative of PG radiosensitivity. Tumours with high PI3K-Akt activation, increased autophagic activity or flux variability, or heightened copper and ROS-handling pathways could derive distinct advantage from PG’s mechanisms. Similarly, metabolic features such as glycolytic predominance or mitochondrial dysfunction could determine sensitivity. Characterising patient-derived three-dimensional organoids or xenografts pre- and post-PG treatment may identify markers of pathway inhibition, DNA damage deposition or GSC pool depletion that correspond to treatment efficacy. Such biosignatures would permit classification of responders and guide personalised therapeutic interventions.

### 8.7 Integrating PG with existing clinical RT protocols

In the case that preclinical evidence supports PG’s radiosensitising potential, the next step would involve conceptual integration with established clinical RT protocols. PG could be assessed along with the Stupp protocol, either as a neoadjuvant agent to prepare tumour cells prior to fractionated RT or as a contemporaneous therapy administered during irradiation (Stupp et al., 2005). Determining optimal sequencing, dosage intervals, and progressive exposure will be crucial, as radiosensitisers often depend on precise temporal arrangements with DNA damage and stress responses induced by radiation. PG and TMZ combination should also be given careful consideration, specifically in MGMT-unmethylated tumours where radiosensitivity is subpar. Intricately designed early-phase trial concepts could evaluate safety, pharmacokinetics, BBB penetration and preliminary indications of improved RT response, providing a framework for future clinical translation.

### 8.8 Radiation-induced immunogenic cell death and the GBM immune microenvironment

A further dimension warranting investigation is whether PG modulates the immune consequences of radiotherapy. Ionising radiation can elicit immunogenic cell death, exposing calreticulin and releasing HMGB1 and ATP that recruit and activate antigen-presenting cells, yet GBM remains an immunologically “cold” tumour that blunts this response. PG drives the same ER-stress, CHOP and autophagic programmes that underlie immunogenic cell death, and has independently been proposed as an immunomodulator capable of reprogramming tumour-associated macrophages, natural killer cells and myeloid-derived suppressor cells within the tumour microenvironment (Anwar et al., 2022). Whether PG amplifies radiation-induced immunogenic cell death or helps convert the cold GBM microenvironment into a radioresponsive one is untested, but it represents a tractable hypothesis–future studies could quantify damage-associated molecular pattern release, dendritic-cell activation and tumour T-cell infiltration in immunocompetent orthotopic GBM models treated with PG and irradiation. This line of enquiry would extend the radiosensitisation rationale beyond direct cytotoxicity to encompass the immune contribution to radiation response.

## 9 Conclusion

Glioblastoma remains profoundly resistant to radiotherapy due to the combined influence of hypoxia, tumour microenvironment dynamics, heightened DNA repair capacity, and the persistence of glioma stem cell populations. Despite incremental advances in multimodal treatment, durable survival gains remain limited, underscoring the need for innovative radiosensitising strategies.

Accumulating preclinical evidence demonstrates that prodigiosin exerts potent antitumour activity in GBM through cytosolic acidification, mitochondrial destabilisation, ER stress induction, autophagy-associated cell death, DNA damage, and modulation of proliferative signalling pathways. These mechanisms intersect with several major determinants of radioresistance, providing a coherent biological rationale for its development as a radiosensitiser.

Recent reviews have catalogued prodigiosin’s anticancer pharmacology in depth, spanning its pro-apoptotic, autophagic and signalling-modulatory activities across a broad range of malignancies (Anwar et al., 2022; Ayoub et al., 2024). None, however, isolates GBM as a single indication, maps PG’s mechanisms onto the determinants of radioresistance, or advances PG as a candidate radiosensitiser. This review departs from that body of work by converting PG’s broad pharmacological profile into a focused, testable proposition–that PG’s mechanistic convergence with the drivers of GBM radioresistance makes it a rational radiosensitiser–and is, to our knowledge, the first to frame the compound specifically against the biology of GBM radiotherapy.

However, the interaction between prodigiosin and ionising radiation has not yet been experimentally defined. Rigorous *in vitro* and *in vivo* studies are needed to determine whether the mechanistic predictions outlined in this review translate into functional radiosensitisation, whether selectivity for tumour over normal brain tissue can be achieved, and how delivery, pharmacokinetics and toxicity constraints may be overcome.

Together, the evidence suggests that prodigiosin represents a compelling yet underexplored candidate for radiosensitisation in GBM. Systematic preclinical investigation, coupled with optimisation of delivery strategies and identification of molecular responders, will be crucial to establishing its translational potential. If effective, prodigiosin could contribute to a new class of multi-mechanistic radiosensitisers capable of addressing several entrenched barriers that currently limit radiotherapy efficacy in GBM.
